# Pediatric Non-Alcoholic Fatty Liver Disease

**DOI:** 10.3390/children4060048

**Published:** 2017-06-09

**Authors:** Haley Bush, Pegah Golabi, Zobair M. Younossi

**Affiliations:** 1 Betty and Guy Beatty Center for Integrated Research, Inova Health System, Falls Church, VA 22042, USA; hbush2@jhu.edu (H.B.); Pegah.Golabi@inova.org (P.G.); 2 Center For Liver Disease, Department of Medicine, Inova Fairfax Hospital, Falls Church, VA 22042, USA

**Keywords:** NAFLD, children, genetics, epidemiology, natural history, obesity, metabolic syndrome

## Abstract

With the increase in the prevalence of obesity, non-alcoholic fatty liver disease (NAFLD) has become among the leading causes of chronic liver disease in the pediatric age group. Once believed to be a “two-hit process”, it is now clear that the actual pathophysiology of NAFLD is complex and involves multiple pathways. Moreover, NAFLD is not always benign, and patients with non-alcoholic steatohepatitis (NASH) are at increased risk of developing advanced stages of liver disease. It has also been shown that NAFLD is not only a liver disease, but is also associated with multiple extrahepatic manifestations, including cardiovascular diseases, type 2 diabetes, and low bone mineral density. Although the data is scarce in the pediatric population, some studies have suggested that long-term mortality and the requirement of liver transplantation will continue to increase in patients with NAFLD. More studies are needed to better understand the natural history of NAFLD, especially in the pediatric age group.

## 1. Introduction

Non-alcoholic fatty liver disease (NAFLD) has emerged as one of the most common chronic liver diseases in the pediatric population, largely due to the rising obesity epidemic in children and adults [1]. NAFLD is currently the second leading indication for liver transplantation in adults in the United States, and is projected to be the most common indication in next decade [2]. Depending on the type of diagnostic tool and the invasiveness of the test, the prevalence of NAFLD in the pediatric population has been estimated to be between 3–12%, but this rate can be as high as 70–80% among those children who are considered obese [3]. Schwimmer et al. identified the prevalence of NAFLD by race and ethnicity among a pediatric population as: Asian: 10.2%, Black: 1.5%, Hispanic: 11.8%, and White: 8.6% [4]. Further research has supported that Hispanic children of Mexican origin have one of the highest incidences of NAFLD, which has been associated with obesity and the *PNPLA3* gene [5]. NAFLD is a metabolic disorder characterized by excessive fat deposition in the liver parenchyma, which is not associated with infection, medication, or an autoimmune process [6]. Additionally, NAFLD encompasses a spectrum of liver diseases, ranging from simple hepatic steatosis, which has also been called NAFL, to non-alcoholic steatohepatitis (NASH), which carries an increased risk of progression to fibrosis, cirrhosis, and even hepatocellular carcinoma [7,8,9]. Despite the plethora of published works examining the epidemiology of NAFLD amongst the pediatric population, the natural history of NAFLD in children is still relatively inadequate due to the lack of long-term prospective studies. Nevertheless, the current evidence suggests that children with NAFLD can develop progressive liver disease and may have shorter survival compared to the unaffected population [10].

The pathogenesis of NAFLD is complex and not fully understood because of the combination of environmental and genetic factors that contribute to the development of NAFLD [11]. Although the initial “two hit” hypothesis was popularized, the current evidence suggests a multi-hit process involving a variety of overlapping pathways. These pathways include lipotoxicity, oxidative stress, gut dysbiosis, endoplasmic reticulum stress, and others [12,13].

Moreover, the development and progression of NAFLD can be influenced by the environment and genetic factors [12,13,14]. One gene in particular, *PNPLA3*, has been associated with NAFLD due to its upregulation in states of overnutrition, association with fat accumulation, and hepatic injury or inflammation [15,16]. Pediatric NAFLD has also been associated with extrahepatic involvement such as the development of atherosclerosis, type 2 diabetes, and low bone mineral density, which makes it a particularly important public health concern [17,18]. In this review, we will discuss the most recent and relevant data regarding the natural history of NAFLD, its pathogenesis and potential genetic modifiers, and we will examine several extrahepatic diseases associated with NAFLD as well as data on their biological mechanisms.

## 2. Natural History of Non-Alcoholic Fatty Liver Disease (NAFLD)

Despite a growing body of knowledge related to NAFLD that has emerged over the last few decades, there is still very limited data providing evidence regarding the natural history of pediatric NAFLD, primarily due to a lack of longitudinal prospective outcomes data. Among the adult population, it is believed that simple hepatic steatosis is generally non-progressive, while NASH may lead to progressive liver disease [19]. In the adult population, the progression of NASH can take years to manifest. In this context, the presence of type 2 diabetes and the severity of histologic features such as ballooning degeneration of hepatocytes can be important predictors of fibrosis progression, but only the stage of fibrosis predicts liver mortality [20]. Similarly, evidence suggests that that among the pediatric population, insulin resistance and obesity are important predictors of progression [21]. In fact, a longitudinal follow-up study on former pediatric NAFLD patients indicated a high rate of type 2 diabetes (30%) and the persistence of obesity into young adulthood [22]. While the exact time of disease onset is still a matter for debate, previous studies have suggested that NAFLD may begin as early as the perinatal period in children of diabetic mothers, and can progress in some children [23]. This evidence originated from three separate studies. The first study examined 25 neonates born to either normal weight (*n* = 13) or obese mothers with gestational diabetes mellitus (*n* = 12) with magnetic resonance imaging for the hepatic fat content at 1–3 weeks of age [24]. The results indicated that infants born to mothers with gestational diabetes had a mean 68% increase in hepatic fat content compared to the infants born to normal weight mothers [24]. The second study reported an increase in abdominal adipose tissue and liver lipids among infants born to mothers with a higher body mass index (BMI) [25]. The last study also examined mother and child pairs and determined that histopathologic hepatic steatosis was significantly more prevalent and severe in stillborn children of diabetic mothers [26].

As noted previously, there is a lack of well-designed prospective natural history studies in children with NAFLD. Nevertheless, Feldstein et al. conducted a retrospective hospital-based cohort study to determine the long-term prognosis of children with NAFLD. This study followed 66 children, with a mean age of approximately 14 years, for a 20-year period. Among the participants, 29% had metabolic syndrome and 83% presented with at least one component of metabolic syndrome (obesity, hypertension, dyslipidemia, and hyperglycemia) [10]. Four children developed type 2 diabetes, two children died, four of 13 histologically re-evaluated patients showed progression of fibrosis, and two underwent liver transplantation for decompensated cirrhosis [10]. In regards to long-term survival, the children with NAFLD had 13.8 times the risk of dying or requiring liver transplantation when compared to the unaffected general population of the same age and sex [10]. This study provides important data suggesting that NASH in children can be progressive, potentially leading to adverse outcomes.

## 3. Pathogenesis and Genetic Modifiers of NAFLD

Pediatric NAFLD occurs in a population of individuals who are overweight and obese, potentially experiencing a variety of metabolic imbalances. Histologically, NAFLD represents an umbrella term that incorporates an entire spectrum of pathologic severity, ranging from steatosis to steatohepatitis with or without fibrosis [27]. While there has been much progress in understanding the pathogenesis of NAFLD, there is still some debate about the pathogenic steps in the progressive form of NAFLD [6]. As previously noted, the “multi-hit hypothesis” seems the most reasonable explanation for the pathogenesis [28]. The “first hit” seems to lead to the intrahepatic accumulation of fatty acids, potentially responsible for insulin resistance and increased vulnerability of hepatocyte to injury [11,28]. The excess of free fatty acids (FFAs) in the liver can also cause hepatic insulin resistance and the collection of incompletely oxidized substrates [29]. One of the “subsequent hits” involves reactive oxygen species (ROS) that can lead to hepatocellular injury by inhibiting the mitochondrial respiratory chain enzymes and cause lipid peroxidation, which can damage hepatocyte membranes [11,28]. It is now clear that a variety of other pathogenic insults may further damage liver cells and activate stellate cells, which are responsible for the formation of collagen, leading to hepatic fibrosis and ultimately cirrhosis [30,31]. Furthermore, while the initial findings regarding fibroblast growth factor (FGF) 21 were contradictory, it has been concluded that serum levels of FGF 21 can be inversely associated with hepatic damage in children with NAFLD [32,33,34]. It is important to recognize some differences in the pathology of NASH between adults and children. In adults, a diagnosis of NASH requires fat accumulation in hepatocytes, inflammation, and liver injury, evident by hepatocyte ballooning [35]. While many of the same histologic features can be identified in the pediatric population, children seem to have more steatosis, fewer ballooned hepatocytes, and more portal-based inflammation [36,37]. These differences in histological findings, particularly the minimal ballooning of hepatocytes and fewer Mallory bodies, classify the pediatric version of NASH as type 2 NASH [38,39,40]. In fact, the initial description defined type 1 NASH as having steatosis, ballooning degeneration, and perisinusoidal fibrosis. Alternatively, type 2 steatosis includes portal inflammation and portal fibrosis [37]. The study concluded that type 1 and type 2 NASH are distinct subtypes of pediatric NAFLD, and that type 2 is the more common amongst children [37].

While obesity and insulin resistance have been identified as some of the greatest risk factors for NAFLD, there are genetic components which have been recognized as possible contributors as well. Due to the complexity of NAFLD pathogenesis, there are a number of genes that contribute to the regulation of a variety of mechanisms such as lipid accumulation in the liver, oxidative stress, inflammation, and fibrogenesis [41,42]. Similar to the data from the adult population, *PNPLA3* has been implicated in children with NASH. This gene was first identified and correlated to NAFLD by Romeo et al. Their study conducted a genome-wide association scan of nonsynonymous sequence variations among 9229 individuals [15]. The *PNPLA3* gene was strongly correlated to increased hepatic fat levels and hepatic inflammation with hepatic content two times greater in homozygote individuals than in non-carriers [15]. Additionally, it was most commonly found amongst Hispanics [15]. In a subsequent study, Schwimmer et al. further supported these findings by describing familial aggregation. In fact, they indicated that fatty liver was significantly more common in siblings (59%) and parents (78%) of children with NAFLD [41]. Furthermore, the available data indicates that heritability estimates range from 20% to 70% depending on the study design, ethnicity, and methodology [43]. While there seems to be a correlation between the *PNPLA3* gene and NAFLD, there has not been sufficient evidence examining its physiological role or mechanism in children with NAFLD.

## 4. Biological Mechanisms and Influences of Extrahepatic Diseases

While NAFLD has been shown to progress to varying degrees of liver damage, this disease has been associated with a number of extra-hepatic diseases. In the adult population, NAFLD has been correlated to having an increased risk of cardiovascular disease (CVD), type 2 diabetes, and more recently chronic kidney disease (CKD) [44,45,46]. There is even evidence to suggest that NAFLD is correlated to sleep apnea, colorectal cancers, osteoporosis, and psoriasis [46,47]. While these extra-hepatic diseases have been more commonly reported in adults with NAFLD, less is known about the strength of their associations and the biological mechanisms involved among the pediatric population with NAFLD.

## 5. Cardiovascular Disease

The relationship between atherosclerosis and NAFLD in children was first identified in a paper by Schwimmer et al. examining 817 children who had died from non-liver causes from 1993 to 2003. The study found that the prevalence of atherosclerosis doubled among children with NAFLD, and the odds of having atherosclerosis were more than six times higher in children with fatty liver compared to those without [48]. Schwimmer et al. also investigated the association between NAFLD and the presence of syndrome in the overweight and obese pediatric population in a case-control study of 150 overweight children with NAFLD and 150 overweight children without NAFLD who were matched for age, sex, and severity of obesity [49]. The children with NAFLD had significantly higher fasting glucose, insulin, total cholesterol, low-density lipoprotein cholesterol, triglycerides, systolic blood pressure, and diastolic blood pressure than overweight and obese children without NAFLD [49]. After additional adjustments for confounders, children with syndrome had five times the odds of also having NAFLD compared to the children without metabolic syndrome [49]. In fact, the association between CVD and NAFLD in children has been further supported by other evidence [50,51]. A review conducted by Pacficio et al. examining the possible biological mechanisms of CVD in NAFLD has suggested insulin resistance, abnormal ectopic fat storage, and low-grade inflammatory state as potential contributors [52]. NAFLD has been associated with an increase in adipose tissue mass and a release of free fatty acids (FFAs), hormones, and proinflammatory adipocytokines where the liver could act as the target organ and source resulting in systemic abnormalities that increase the risk of developing CVD [52,53,54]. The increased levels of FFAs could prompt myocardial lipid accumulation with a negative effect on left ventricle function in the heart [52,55,56,57,58]. Lastly, Gaudio et al. proposed that macrophage infiltration can cause systemic inflammatory signals, which can lead to the progression of both atherosclerotic plaques and steatohepatitis [52,59].

## 6. Type 2 Diabetes

An abstract presented by Lavine et al. examined the natural history of NASH among a group of 57 children and determined that within the two years of study follow-up time, 10% of these children had developed diabetes [17,60]. It is important to note that in adults with NAFLD, type 2 diabetes has been a risk factor for increased mortality [61,62]. In children, there are no long-term studies of NAFLD and type 2 diabetes. In one study, Persighin et al. examined 54 obese adolescents, 16 of which had a diagnosis of NAFLD. The NAFLD subjects had higher fasting plasma glucose and impaired insulin sensitivity [63]. Furthermore, two separate studies identified the correlation between fatty liver and peripheral muscle insulin sensitivity [64,65]. Additionally, Patton et al. identified that some individual metabolic syndrome features, central obesity and insulin resistance, are related to pediatric NAFLD severity. The same study analyzed 254 children for the frequency and distribution of individual components of metabolic syndrome central obesity (67%), high triglycerides (26%), low level of high-density lipoprotein cholesterol (26%), hypertension (45%), and impaired fasting glucose (12.2%) [66]. Although the exact mechanism is not fully understood, there have been several proposed biological mechanisms for the progression to type 2 diabetes. One study suggests that B-cells in subjects with NAFLD are unable to adequately compensate for the ambient level of insulin resistance, which increases the susceptibility to type 2 diabetes [64]. Several studies have emphasized the importance of low adiponectin levels in obese adolescents as a biomarker for type 2 diabetes and possibly NASH, which may warrant early intervention [67,68]. In contrast, another study highlighted the importance of a low-grade inflammatory state and inflammatory proteins (tumor necrosis factor (TNF), interleukin (IL)-6, leptin, and resistin), which are generated by visceral adipose tissue and enter the portal vein, a pathway that has been connected to the pathogenesis of both NASH and insulin resistance [62,69].

## 7. Low Bone Mineral Density

Paredee et al. conducted a study to determine the association between bone mineral density (BMD) in obese children with and without NAFLD. The study included 38 children with a diagnosis of NAFLD who were matched for age, gender, race, ethnicity, height, and weight to children without NAFLD [18]. It was determined that 45% of the children with NAFLD had low BMD in comparison to none of the controls [18]. The specific biological mechanisms underlying the correlation between NAFLD and low BMD have not been properly established, but there are multiple hypotheses related to potential inflammatory responses. Several studies have established the involvement of systemic inflammation in low BMD [70,71,72]. It has been shown that (TNF-α) increases osteoclast activity, inhibits osteoblast differentiation, and enhances osteoblast apoptosis [73,74]. These cytokines and regulation pathways have been associated with the development of NAFLD [75]. Therefore, it is thought that the presence of systematic and constant inflammation in NAFLD patients contributes to the correlation between NAFLD and low BMD.

## 8. Summary

There is a growing epidemic of obesity and its complications, including NAFLD, among children. Pediatric NAFLD is a complex chronic liver disease with a number of contributing factors. Currently, there is some evidence supporting the association between pediatric NAFLD with the development of cardiovascular disease, type 2 diabetes, and low bone mineral density. Despite these associations, the pathogenic mechanisms are not always clear and further research is required to shed light on the pathways involved in these extrahepatic manifestations. It is clear that there is a tremendous scarcity of good data regarding the natural history of NASH in children. Although the treatment of obesity is the most logical first step in treating children with NASH, other treatments are not available. The rapidly growing prevalence of pediatric NAFLD and its potential for progression to advanced liver could potentially result in a tsunami of NAFLD-related liver diseases and an economic burden to the society in the future [76]. In this context, it is critical that this complex liver disease should be addressed through collaborations among healthcare providers, public health experts, policymakers, and the healthcare industry to develop a comprehensive multi-year strategy to manage this important complication of obesity. However, this requires a national policy and funding for translational and outcomes research.
